# Impact of *TP53* Codon 72 and *MDM2* SNP 309 Polymorphisms in Pancreatic Ductal Adenocarcinoma

**DOI:** 10.1371/journal.pone.0118829

**Published:** 2015-03-03

**Authors:** Yasuki Hori, Katsuyuki Miyabe, Michihiro Yoshida, Takahiro Nakazawa, Kazuki Hayashi, Itaru Naitoh, Shuya Shimizu, Hiromu Kondo, Yuji Nishi, Shuichiro Umemura, Akihisa Kato, Hirotaka Ohara, Hiroshi Inagaki, Takashi Joh

**Affiliations:** 1 Department of Gastroenterology and Metabolism, Nagoya City University Graduate School of Medical Sciences, Nagoya, Japan; 2 Department of Community-based Medical Education, Nagoya City University Graduate School of Medical Sciences, Nagoya, Japan; 3 Department of Pathology and Molecular Diagnostics, Nagoya City University Graduate School of Medical Sciences, Nagoya, Japan; University of Saarland Medical School, GERMANY

## Abstract

Single-nucleotide polymorphisms (SNPs) of *TP53* (codon 72, rs1042522) and *MDM2* promoter (SNP 309, rs2279744) have been associated with risk for various human cancers. However, studies analyzing these polymorphisms in pancreatic ductal adenocarcinoma (PDAC) are lacking. We investigated *TP53* codon 72 and *MDM2* SNP 309 polymorphisms in 32 patients with PDAC, 16 patients with chronic pancreatitis (CP), and 32 normal controls, using formalin-fixed paraffin-embedded tissue. We also examined *TP53* and *MDM2* protein immunohistochemistry (IHC) to assess the involvement of these differences in malignant transformation and disease progression. *TP53* Pro/Pro genotype was significantly more frequent in PDAC patients than in controls (65.6 *vs*. 15.6%, *p* < 0.001) and no significant difference was found between CP patients (37.5%) and controls. In *MDM2* SNP 309, there were no significant differences among the three groups. Based on the Kaplan-Meier analysis, overall survival was significantly shorter in *MDM2* G/G genotypes compared with other genotypes (G/T and T/T) (359 *vs*. 911 days, *p* = 0.016) whereas no significant differences in *TP53* genotypes were observed (638 *vs*. 752 days, *p* = 0.471). Although *TP53* IHC was frequent in PDAC patients (53.1%), *TP53* and *MDM2* protein expression was not correlated with polymorphisms. Our study demonstrated *TP53* codon 72 polymorphism is potentially a genetic predisposing factor while *MDM2* SNP 309 polymorphism might be useful in predicting survival outcome.

## Introduction

Pancreatic ductal adenocarcinoma (PDAC) is a gastrointestinal neoplasm with high malignancy and poor prognosis. Incidence of PDAC has increased in recent years, but the therapeutic efficacy remains unsatisfactory. Complete surgical resection is an essential part of curative therapy. However, most tumors are unresectable and are treated primarily with chemotherapy and/or radiation [1–3]. Recent research has shown that single nucleotide polymorphisms (SNPs) of genes involved in the cell cycle play an important role in carcinogenesis [4–7] and that common polymorphisms may lead to altered susceptibility to PDAC and affect clinical outcome [7].

The tumor protein *p53* (*TP53*) tumor suppressor pathway plays a critical role in cell cycle regulation and apoptosis in many cancers, including PDAC. A common polymorphism located in exon 4 of *TP53* gene, resulting in a non-conservative arginine (Arg) to a proline (Pro) change at codon 72, is important for growth suppression and apoptotic function [8]. Additionally, the T to G allelic change introduced by *mouse double minute 2* (*MDM2*) promoter SNP 309 was predicted to increase the affinity of the Sp1 transcription factor by extending the length of a Sp1 binding site, resulting in repressed tumor suppressor activity of the p53 pathway [4]. This finding was subsequently validated through *in vitro* DNA-protein binding and reporter plasmid cell-based assays [4]. These polymorphisms were investigated previously in peripheral blood samples from PDAC patients [9–11]. To our knowledge, in PDAC, no study has examined *TP53* codon 72 and only one study has examined the *MDM2* SNP 309 [12] polymorphism using formalin-fixed paraffin-embedded (FFPE) specimens. Furthermore, the relationship between these polymorphisms and malignant transformation has not been investigated in chronic pancreatitis (CP), which is considered a precancerous change.

Immunohistochemistry (IHC) is a method widely used for investigation of TP53 protein expression [13]. TP53 nuclear accumulation is considered a result of *TP53* stabilization either by a mutation or cellular stresses. *MDM2* is a downstream gene and its expression is induced by wild-type *TP53*. However, the expression of MDM2 protein can also occur independently of *TP53*, even if its stress induction is *TP53*-dependent. Therefore, IHC of MDM2 was suggested as a possible method to discriminate between functional and nonfunctional *TP53* in human tumors [14].

In the present study, using IHC we examined the *TP53* codon 72 and *MDM2* SNP 309 polymorphisms and TP53 and MDM2 proteins in PDAC and CP patients to assess the involvement of these differences in the malignant transformation and disease progression.

## Materials and Methods

### Study population and samples

Between January 1997 and December 2010, PDAC patients and CP patients were retrospectively evaluated. We also cases of pancreatic epithelium from resected specimens without pancreatic disease as controls (normal controls). All cases were obtained from the archives of the Department of Pathology, Nagoya City University Graduate School of Medical Sciences and affiliated hospitals. The CP cases consisted of both alcoholic and idiopathic CP and were diagnosed based on the Zurich classification [15] including history of excessive alcohol intake (in excess of 80 g ethanol per day), calcification in the pancreas, or moderate to marked ductal lesions described in the Cambridge classification [16]. CP pathology was characterized by perilobular fibrosis and acinar destruction with acute and chronic inflammatory cells.

In order to determine the sample size, we conducted an interim analysis of TP53 IHC in 32 cases of PDAC and 21 normal controls. TP53 IHC was positive in 17 of the 32 PDAC patients (53.1%) and 3 of 21 normal controls (14.3%). For a 5% type I error with 80% statistical power, the required number of patients in each group was estimated to be 28. Therefore, additional normal controls gave 32 normal controls. All of the available CP cases were collected (n = 16).

This study was approved by the Review Board of Nagoya City University Graduate School of Medical Sciences (approval No. 990).

### DNA isolation and PCR amplification of *TP53* codon 72 and *MDM2* SNP 309 polymorphisms

Formalin-fixed paraffin-embedded tissue blocks were used for DNA extraction for gene amplification. Tumor samples were macrodissected from FFPE tissues blocks guided with hematoxylin-and-eosin (H&E) stained sections. Following deparaffinization with xylene and alcohol, genomic DNA was extracted using the QIAamp DNA Mini Kit (QIAGEN, Valencia, CA, USA).

The status of *TP53* codon 72 (rs1042522) and *MDM2* SNP 309 (rs2279744) was determined in the study participants using a TaqMan SNP Genotyping Assay. Primers were purchased from Applied Biosystems (Foster City, CA, USA). Assay ID for *TP53* codon 72 (rs1042522) was C_2403545_10. Primers and probes for *MDM2* SNP 309 were as follows: 5′-GACTACGCGCAGCGTTCA-3′ (forward); 5′-AGGTCTCCGCGGGAGTTC-3′ (reverse); 5′-CGCGCCGCAGCGGC-3′ (VIC); 5′-CCGCGCCGAAGCGGC-3′ (FAM).

Genotyping was performed on an Applied Biosystems 7500 FAST Real-Time PCR System using a TaqMan SNP genotyping assay (Affymetrix Inc., Cleveland, OH, USA). Each reaction (10 μL) contained 5 μL TaqMan Genotyping Master Mix, 0.5 μL primers and probes (Applied Biosystems), 3.5 μL water and 1 μL DNA (5–10 μL/μL). Thermal cycling conditions were 95°C for 10 min, followed by 50 cycles of 95°C for 15 sec and 60°C for 1 min.

### IHC staining

The same tissue block used for extracting genomic DNA was selected from each case slide. Serial 3-μm thick sections were made from FFPE tissue blocks.

Tissue sections were deparaffinized and rehydrated. After heat-induced antigen retrieval, IHC was performed using an automated immunostainer (Bond-Max, Leica MicroSystems, Wetzlar, Germany) with monoclonal antibodies against TP53 (clone DO7; dilution, 1:200) and MDM2 (clone 1B10; 1:200). Nuclei were counterstained with hematoxylin. Nuclear staining of TP53 and MDM2 protein was shown as brown granules. A positive result was defined as more than 30% of the tumor cells showing positive staining.

All slides were reviewed in a blinded manner without clinical information by two independent authors (H. I. and K.M.). When the assessment was different between the two observers, agreement was reached using a double-headed microscope. These slides were observed using a light microscope (Nikon ECLIPSE 80i, Nikon Corporation Tokyo, Japan) with a 40× field objective and 10× ocular lens corresponding to a field diameter of 550 μm for the slides.

### Statistical analyses

The chi-square test and Fisher’s exact test were used to assess the significance of any difference in the prevalence of *TP53* codon 72 and *MDM2* SNP 309 polymorphisms among PDAC, CP and normal control groups. Tests for Hardy—Weinberg equilibrium were conducted using a goodness-of-fit chi-square test to compare the observed genotype frequencies with the expected genotype frequencies using reported frequencies in Japanese populations with two degree of freedom. The odds ratio (OR) and 95% confidence intervals (CI) were used as measures of the association strength. Values of *p* ≤ 0.05 without the Bonferroni correction and *p* ≤ 0.017 with the Bonferroni correction (two comparisons between three groups) were considered statistically significant. Kaplan—Meier analysis was used to analyze overall survival period. A Cox proportional hazards analysis was also performed to identify factors that could lead to a shorter overall survival period.

## Results

### Patient characteristics (Table 1)

This study included 32 PDAC patients, 16 CP patients and 32 normal controls (median age, 64 years; range, 32–82 years). The patient characteristics are summarized in Table 1. No significant differences in the distribution of gender, age, body mass index (BMI), smoking, or diabetes were observed among three groups. Among PDAC patients, 14 patients (43.8%) received chemotherapy, 2 (6.3%) received radiotherapy and 5 (15.6%) received chemoradiotherapy after tumors were resected. The follow-up period ranged from 3.1 to 101.7 months (median, 23.1 months). The overall one-year survival rate was 71.9% (23/32).

**Table 1:** Patient characteristics.

PDAC	n = 32
Gender (Male/Female)	24/8
Age (median) [range]	68 [32–82]
BMI (average) [range]	20.6 [16.0–24.9]
Smoking (Yes/No)	16/16
Diabetes (Yes/No)	10/22
Pancreatitis (Yes/No)	2/30
Location (head/body and tail)	18/14
TNM stage (I/II/III/IV)	2/3/15/12
Grade (well/moderate/poor)	19/11/2
Residual (R0/R1)	27/5
Adjuvant therapy/ No adjuvant therapy(chemotherapy/radiotherapy/chemo-radiotherapy)	21/11(14/2/5)

CP	n = 16
Gender (Male/Female)	15/1
Age (median) [range]	54.5 [37–70]
BMI (average) [range]	21.3 [18.3–23.6]
Smoking (Yes/No)	12/4
Diabetes (Yes/No)	4/12
Alcohol (Yes/No)	14/2

Normal	n = 32
Gender (Male/Female)	23/9
Age (median) [range]	64.5 [43–84]
BMI (average) [range]	21.6 [16.8–24.7]
Smoking (Yes/No)	16/16
Diabetes (Yes/No)	7/25

### Genotype frequency in each group

**Fig 1:**
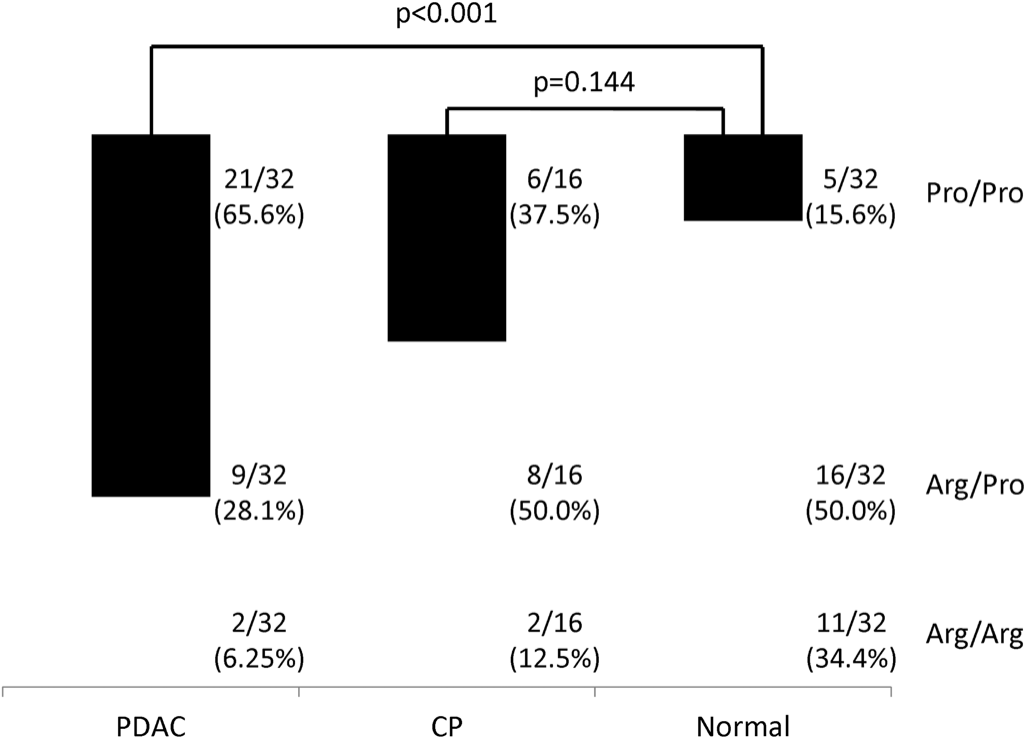
Distribution of *TP53* codon 72 genotypes among pancreatic ductal adenocarcinoma (PDAC) patients, chronic pancreatic (CP) patients and normal controls. The *p*-value was calculated by comparing the Pro/Pro genotype between PDAC patients and normal controls and between CP patients and normal controls.

**Fig 2:**
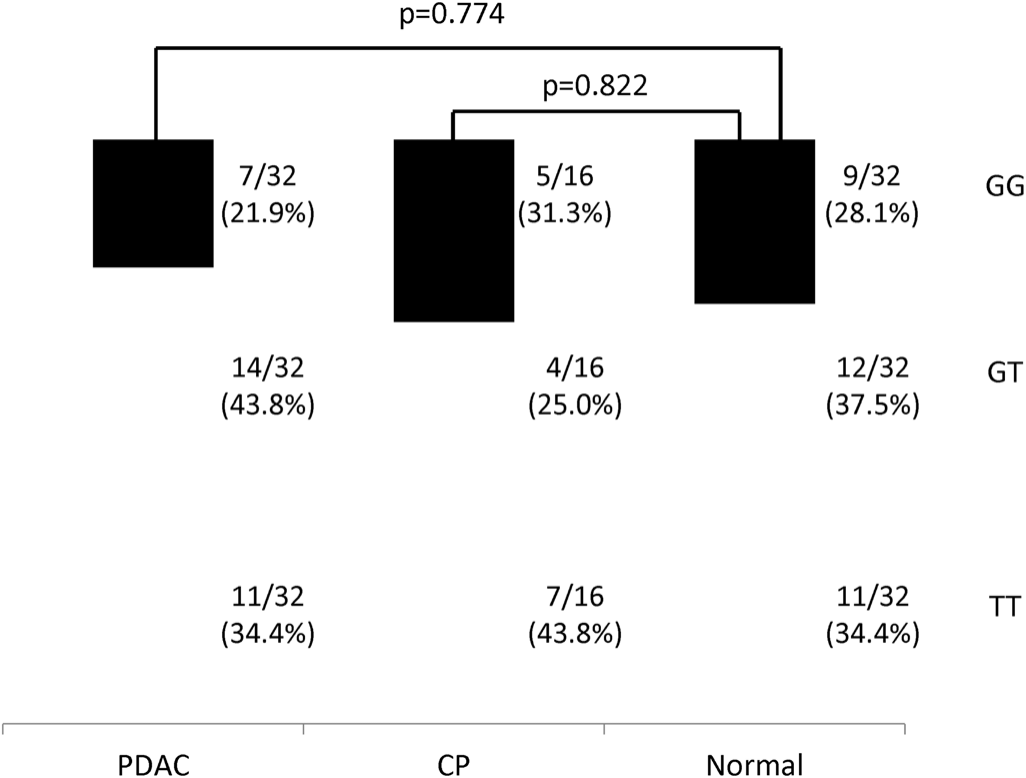
Distribution of *MDM2* single-nucleotide polymorphism (SNP) 309 genotypes among pancreatic ductal adenocarcinoma (PDAC) patients, chronic pancreatitis (CP) patients and normal controls. The *p*-value was calculated by comparing the G/G genotype between PDAC patients and normal controls and between CP patients and normal controls.

Detection of *TP53* codon 72 and *MDM2* SNP 309 polymorphisms using real—time PCR was conducted successfully in all cases. Distributions of these polymorphisms in patients and controls are shown in Figs. 1 and 2.

The genotype distribution of the *TP53* codon 72 polymorphism was as follows: 21 cases (65.6%) of Pro/Pro, 9 (28.1%) of Arg/Pro and 2 (6.25%) of Arg/Arg genotypes in PDAC; 6 (37.5%) cases of Pro/Pro, 8 (50%) of Arg/Pro and 2 of (12.5%) Arg/Arg in CP; 5 (15.6%) of Pro/Pro, 16 (50.0%) of Arg/Pro and 11 (34.4%) of Arg/Arg in normal controls. The Pro/Pro genotype was more frequent in PDAC patients than in normal controls (*p* < 0.001; adjusted OR, 10.31; 95% CI, 3.17–33.24), whereas no significant difference of Pro/Pro frequency was observed between CP patients and controls (*p* = 0.144).

Conversely, *MDM2* SNP 309 polymorphism showed different genotype distribution in each group as follows: 7 cases (21.9%) of GG, 14 (43.8%) of GT and 11 (34.4%) of TT genotypes in PDAC; 5 cases (31.3%) of GG, 4 (25%) of GT and 7 (43.8%) of TT in CP; 9 (28.1%) of GG, 12 (37.5%) of GT and 11 (34.4%) of TT in normal controls. The genotype frequency was not significantly different among the three groups (PDAC *vs*. normal, *p* = 0.774; CP *vs*. normal, *p* = 0.822).

The genotype frequencies reported in articles studying Japanese populations fit Hardy—Weinberg equilibrium (HWE, Table 2, 3). Therefore, we used a pooled control group reported previously for the normal Japanese population in order to investigate HWE. This step increased the number of control cases for better comparison with our results. The distribution of the TP53 codon 72 polymorphism in normal controls (*χ* ^*2*^ = 0.571, df = 2, *p* = 0.752) fit HWE, while those in PDAC (*χ* ^*2*^ = 78.95, df = 2, *p* < 0.001) and CP (*χ* ^*2*^ = 10.44, df = 2, *p* = 0.005) did not fit HWE (Table 4). The distribution of the *MDM2* SNP 309 polymorphism in PDAC (*χ* ^*2*^ = 2.155, df = 2, *p* = 0.340), CP (*χ* ^*2*^ = 4.944, df = 2, *p* = 0.084) and normal controls (*χ* ^*2*^ = 2.658, df = 2, *p* = 0.265) fit HWE (Table 5).

**Table 2:** *TP53* codon72 genotypes of normal controls in Japanese populations.

			*TP53* codon72 genotype			HWE		MAF
Author	Year	Sample size	Pro/Pro	Pro/Arg	Arg/Arg	Chi-square	*P* value	
Sakiyama *et al*. [33]	2005	685	73	310	302	0.247	0.619	0.333
Kiyohara *et al*. [34]	2010	379	42	175	162	0.264	0.607	0.342
Horikawa *et al*. [35]	2008	267	38	136	93	1.089	0.297	0.397
Kuroda *et al*. [36]	2003	175	35	77	63	1.643	0.200	0.420
Wu *et al*. [37]	1995	56	6	24	26	0.017	0.896	0.321
Kuroda *et al*. [38]	2007	271	45	117	109	1.982	0.159	0.382
Takeuchi *et al*. [39]	2005	89	20	37	32	2.087	0.149	0.433
Hishida *et al*. [40]	2004	440	56	199	185	0.048	0.827	0.353
Mabuchi *et al*. [41]	2009	189	23	83	83	0.102	0.749	0.341
Joshi *et al*. [42]	2011	778	107	361	310	0.014	0.906	0.370
Yoneda *et al*. [43]	2013	200	23	102	75	1.765	0.184	0.370
Total								
		3529	468	1621	1440	0.019	0.890	0.362

Pro, proline; Arg, arginine; HWE, Hardy-Weinberg equilibrium; MAF, minor allele frequency

**Table 3:** *MDM2* SNP309 genotypes of normal controls in Japanese populations.

			*MDM2* SNP309 genotype			HWE		MAF
Author	Year	Sample size	G/G	G/T	T/T	Chi-square	*P* value	
Horikawa *et al*. [35]	2008	266	79	132	55	0.000	1	0.455
Nakashima *et al*. [44]	2008	120	33	50	37	3.296	0.069	0.483
Joshi *et al*. [42]	2011	778	217	384	177	0.082	0.775	0.474
Sugano *et al*. [45]	2010	59	20	27	12	0.270	0.603	0.432
Yoneda *et al*. [43]	2013	200	40	98	62	0.013	0.909	0.445
Total								
		1423	389	691	343	1.099	0.294	0.484

HWE, Hardy-Weinberg equilibrium; MAF, minor allele frequency

**Table 4:** *TP53* codon72 genotypes and HWE.

		*TP53* codon72 genotype			HWE		MAF
	Sample size	Pro/Pro	Pro/Arg	Arg/Arg	Chi-square	*P* value	
PDAC	32	21	9	2	78.95	<0.001	0.203
CP	16	6	8	2	10.44	0.005	0.375
Normal	32	5	16	11	0.571	0.752	0.406

Pro, proline; Arg, arginine; HWE, Hardy-Weinberg equilibrium; MAF, minor allele frequency

**Table 5:** *MDM2* SNP309 genotypes and HWE.

		*MDM2* SNP309 genotype			HWE		MAF
	Sample size	G/G	G/T	T/T	Chi-square	*P* value	
PDAC	32	7	14	11	2.155	0.340	0.438
CP	16	5	4	7	4.944	0.084	0.438
Normal	32	9	12	11	2.658	0.265	0.469

HWE, Hardy-Weinberg equilibrium; MAF, minor allele frequency

### Genotype effect on survival

**Fig 3:**
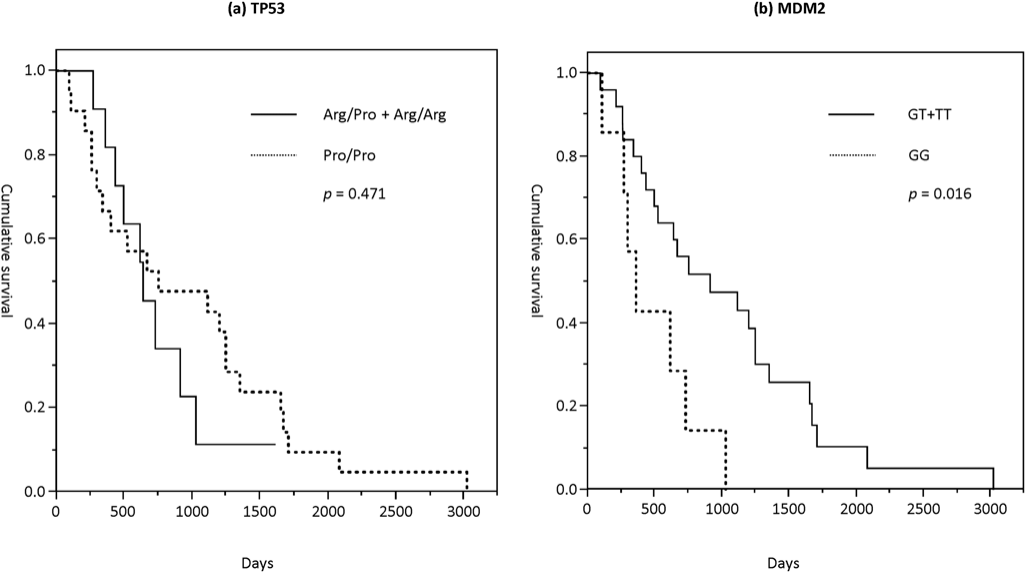
The overall survival based on genotype in pancreatic ductal adenocarcinoma (PDAC) patients using Kaplan-Meier analysis. **(a)** *TP53* codon 72 genotypes **(b)** *MDM2* single-nucleotide polymorphism (SNP) 309 genotypes.

Overall survival data of all PDAC patients were included in the survival analysis. Based on the log-rank test and Kaplan-Meier survival curve analyses, overall survival was not significantly different between Pro/Pro and other genotypes (*p* = 0.471; Fig. 3a). The median survival of patients with Pro/Pro and other genotypes was 638 days (95% CI, 504–978 days) and 752 days (631–1291 days), respectively.

By contrast, the median survival of *MDM2* G/G genotype and other genotypes (T/G and T/T) were 359 days (249–722 days) and 911 days (732–1333 days), respectively. Overall survival was significantly shorter in the G/G genotypes compared with other genotypes (*p* = 0.016, Fig. 3b).

### IHC of TP53 and MDM2 for polymorphism comparison

**Fig 4:**
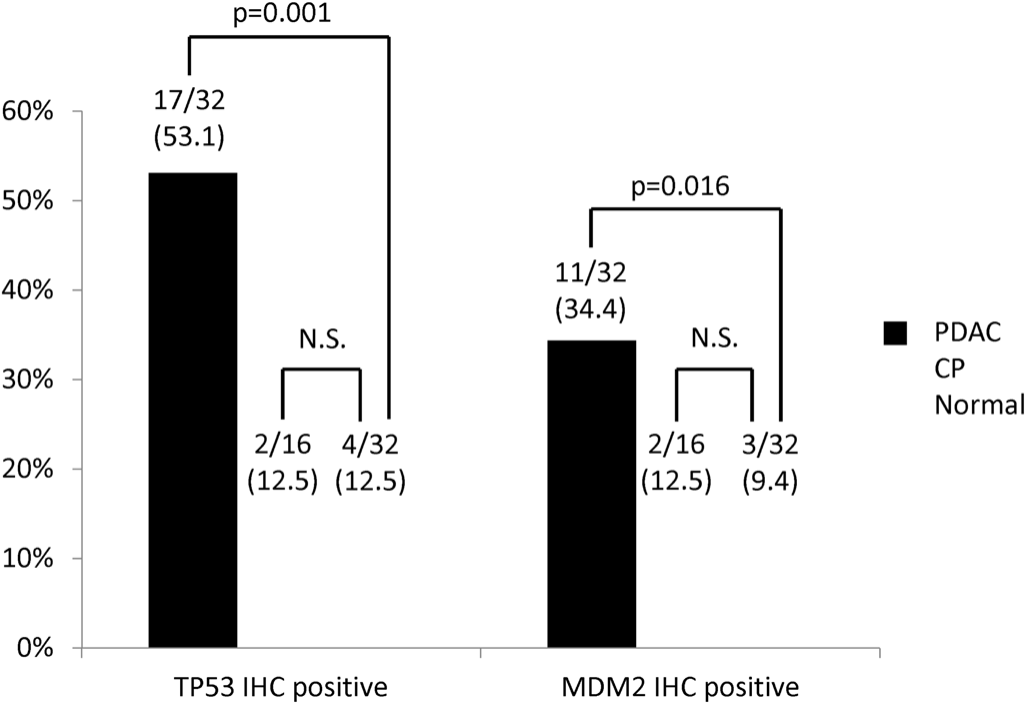
Clinicopathological correlations of TP53 and MDM2 protein expression in pancreatic ductal adenocarcinoma (PDAC) patients, chronic pancreatitis (CP) patients and normal controls.

As shown in Fig. 4, TP53 IHC was positive in 17 of 32 PDAC patients (53.1%), 2 of 16 CP patients (12.5%) and 4 of 32 normal controls (12.5%). MDM2 IHC was positive in 11 of 32 PDAC patients (34.4%), 2 of 16 CP patients (12.5%) and 3 of 32 normal controls (9.4%). The positive expression rate of TP53 IHC was significantly more frequent in PDAC than in normal controls (*vs*. normal controls, *p* = 0.001) and the positive expression rate of MDM2 IHC was significantly more frequent in PDAC than in normal controls (*p* = 0.016). The positive expression rates of TP53 and MDM2 IHC in CP patients did not significantly differ from the rate in normal controls.

**Fig 5:**
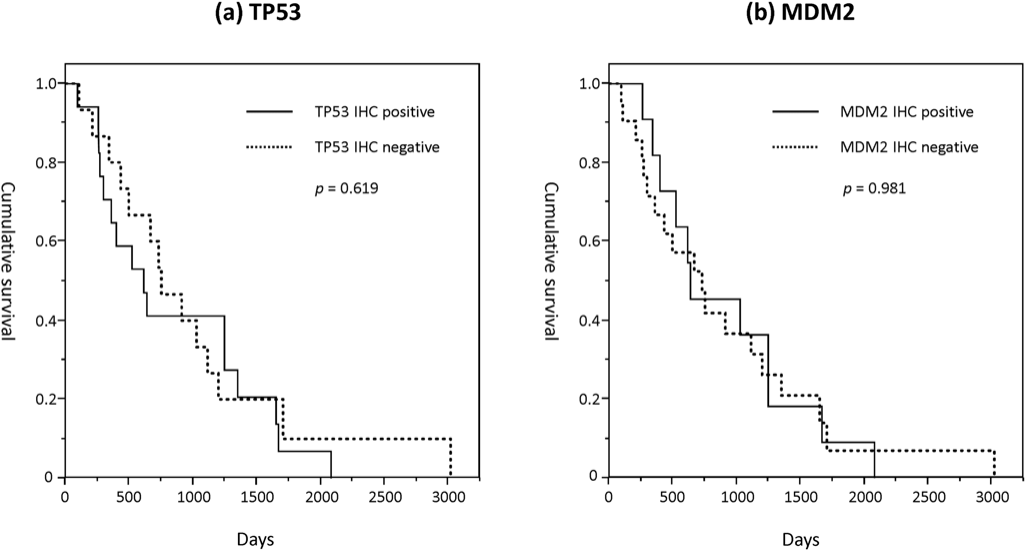
Kaplan-Meier analysis of the overall survival using immunohistochemistry (IHC) results in pancreatic ductal adenocarcinoma (PDAC) patients. **(a)** TP53 IHC positive/negative, **(b)** MDM2 IHC positive/negative.

Although *MDM2* G/G genotypes and overall survival were associated, no statistically significant differences of overall survival were observed between IHC-positive and IHC-negative PDAC patients (TP53 IHC positive *vs*. negative, *p* = 0.619; MDM2 IHC positive *vs*. negative, *p* = 0.981, Fig. 5a, b).

### Multivariate analysis adjusted by potential confounding factors

The Cox proportional hazards model was applied to multifactor analysis using the following variables: age (<60 or ≥60 years), gender, smoking history, body mass index (BMI, <20 or ≥20), TNM staging, *TP53* codon 72 genotype (Pro/Pro or not), *MDM2* SNP 309 genotype (G/G or not), IHC of TP53 and MDM2, and the existence of tumor-node-metastases. The outcomes indicated that all factors, except the *MDM2* G/G genotype, were not correlated with overall survival.

## Discussion

Polymorphism in *TP53* codon 72 produces two different P53 proteins because of a single base change altering CGC to CCC in the fourth exon of the *TP53* gene, altering amino acid residue 72 from Arg to Pro [17, 18]. An association of the *TP53* codon 72 polymorphism with several cancer susceptibilities has been reported [19–29]. Additionally, a common *MDM2* promoter polymorphism is the T→G transformation at nucleotide 309. This *MDM2* 309T/G promoter polymorphism is associated with the development of a variety of tumors [4–6]. However, its association with PDAC has not been fully evaluated. Therefore, we investigated the involvement of *TP53* and *MDM2* in malignant transformation and disease progression. To our best knowledge, this is the first study evaluating the significance of *TP53* codon 72 polymorphism using FFPE pancreatic tissue.

We confirmed that the ratio of Pro/Pro genotype was significantly more frequent in PDAC patients than in controls, even using FFPE tissues, and the finding supports the hypothesis that this polymorphism influences the *TP53* gene expression. In this study, the normal controls satisfied HWE, as compared with a pooled analysis from previous articles, which indicates that they were concurrent with the general population. This result vindicates the statistical outcomes in the study. Furthermore, the Pro/Pro frequency in PDAC was higher in this study using FFPE tissues (65.6%) than in the studies of Sonoyama *et al*. (14.6%) [9] or Nacaeeati *et al*. (7.9%) [30] using blood samples. One of the plausible explanations for this finding is the influence of FFPE; however, HWE in normal controls with FFPE in this study suggests that it is unlikely. Another plausible explanation is the occurrence of a somatic mutation that is also supported by the failure to fit HWE in PDAC with FFPE tissues. The *TP53* codon72 somatic mutation might play an important role in carcinogenesis or cancer progression of PDAC, as well as its germline mutation [31].

In CP, the presence of the *TP53* codon 72 allele was not statistically significant. In a previous study, when using tissues obtained by endoscopic ultrasound-guided fine needle aspiration (EUS-FNA), the *K-ras* mutation status was considered a biomarker for PDAC [32]. Similarly, this finding, combined with the conventional cytology test, might provide more accurate diagnosis to distinguish PDAC from CP such as mass-forming pancreatitis, using only a small specimen obtained by EUS-FNA.

The difference of the *MDM2* SNP 309 among the three disease groups was not statistically significant. The HWE of *MDM2* SNP 309 in PDAC, CP, and normal controls indicates that few cases have a somatic mutation at this site. However, the analysis of overall survival revealed significant differences between the GG allele and GT/TT allele of SNP 309 genotypes, which was not obtained in the analysis of the *TP53* codon 72 genotypes. Our results are consistent with a growing body of evidence supporting a deleterious role of the G allele in this polymorphism on disease outcome. The G allele is associated with increased affinity for Sp1 binding and higher *MDM2* RNA and protein levels, weakening the role of the *TP53* pathway [4]. Another study suggested the G allele of the *MDM2* SNP 309 T/G polymorphism is associated with increasing risk and progression of PDAC and corresponding decrease in survival [10], which partially supports our data.

We also evaluated and compared TP53 and MDM2 protein expressions using IHC on the outcome of SNP genotypes. Our results confirmed that the positive expression rate of TP53 and MDM2 protein was significantly more frequent in PDAC than in CP patients and normal controls, and that the rate in CP was similar to that of the normal controls. Positive TP53 and MDM2 protein expression in PDAC patients was similar to previous reports [5], indicating that our findings in the present study were reliable. Furthermore, the similar distribution of IHC expression in CP patients and normal controls suggest these protein expressions in CP patients were different from PDAC patients. Therefore, based on these protein expressions, CP is not a precancerous entity.

In conclusion, our study demonstrated that the *TP53* codon 72 polymorphism is potentially a genetic predisposing factor for pancreatic cancer and *MDM2* SNP 309 polymorphism might be useful for predicting survival outcome in PDAC patients. These findings provide insights into the oncogenesis and molecular diagnosis of PDAC.

## Supporting Information

S1 Table Raw data in our cases. BI, Brinkman Index; BW, body weight; BMI, body mass index.
(XLSX)
