# Correction: Impact of *TP53* Codon 72 and *MDM2* SNP 309 Polymorphisms in Pancreatic Ductal Adenocarcinoma

**DOI:** 10.1371/journal.pone.0126295

**Published:** 2015-04-13

**Authors:** The PLOS ONE Staff

Fig 1, Fig 2, and Fig 4 appear incorrectly in both the HTML and PDF versions of this article. Please view the corrected versions of Fig 1, Fig 2, and Fig 4 here.

**Fig 1:**
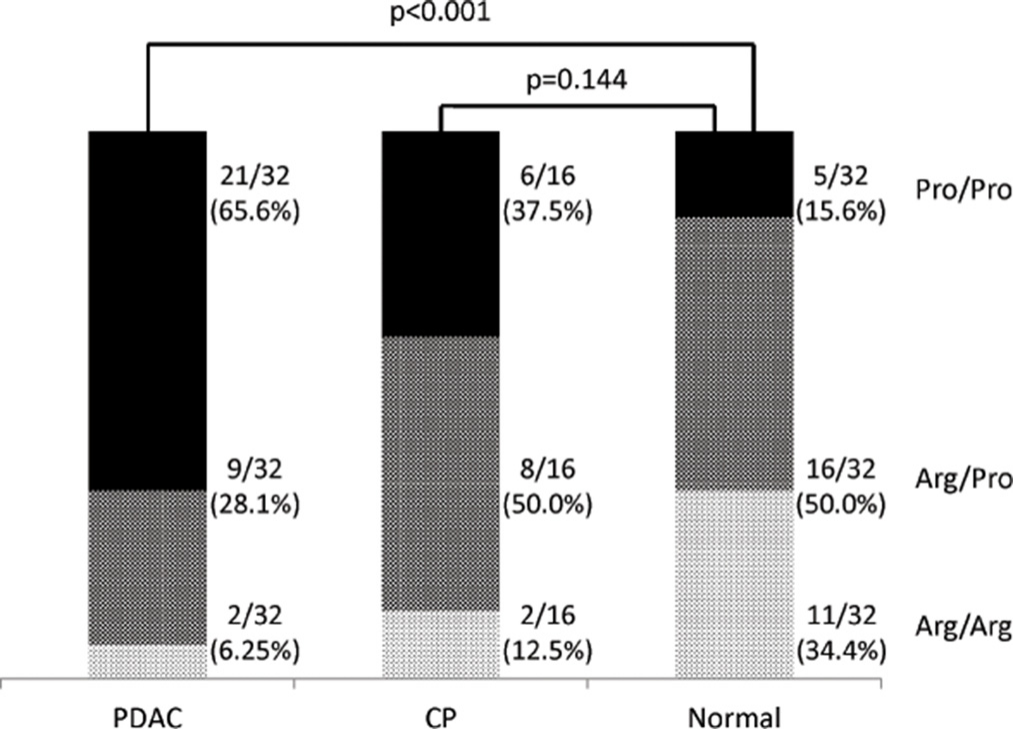
Distribution of *TP53* codon 72 genotypes among pancreatic ductal adenocarcinoma (PDAC) patients, chronic pancreatic (CP) patients and normal controls. The *p*-value was calculated by comparing the Pro/Pro genotype between PDAC patients and normal controls and between CP patients and normal controls.

**Fig 2:**
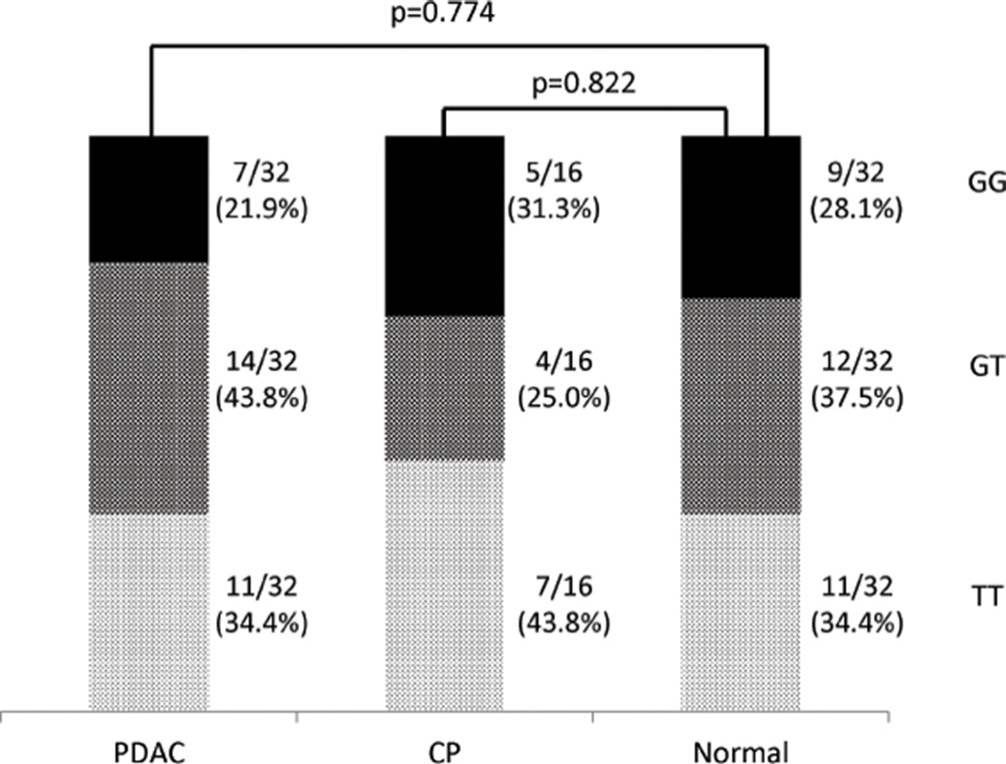
Distribution of *MDM2* single-nucleotide polymorphism (SNP) 309 genotypes among pancreatic ductal adenocarcinoma (PDAC) patients, chronic pancreatitis (CP) patients and normal controls. The *p*-value was calculated by comparing the G/G genotype between PDAC patients and normal controls and between CP patients and normal controls.

**Fig 4:**
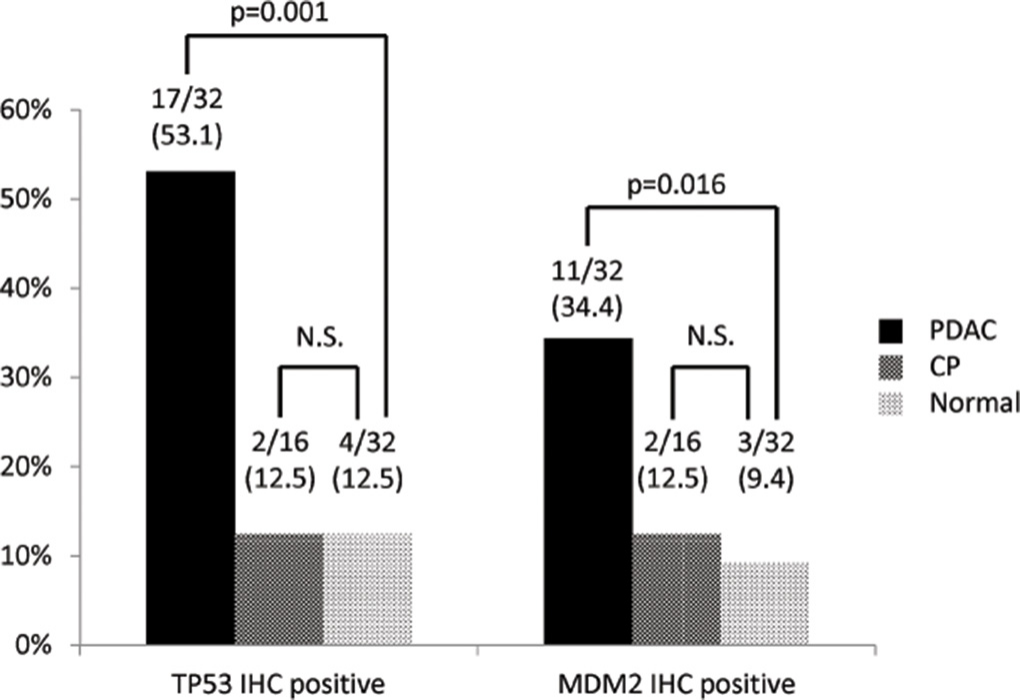
Clinicopathological correlations of TP53 and MDM2 protein expression in pancreatic ductal adenocarcinoma (PDAC) patients, chronic pancreatitis (CP) patients and normal controls.
